# Intracerebral hemorrhage as a complication of reversible cerebral vasoconstriction syndrome in the postpartum periods: a case report

**DOI:** 10.11604/pamj.2024.48.164.44737

**Published:** 2024-08-08

**Authors:** Nazla Ananda Rachmi Puti, Achmad Firdaus Sani, Dedy Kurniawan

**Affiliations:** 1 Department of Neurology, Faculty of Medicine, Universitas Airlangga, Soetomo Academic General Hospital, Surabaya, Indonesia,

**Keywords:** Reversible cerebral vasoconstriction syndrome, postpartum, nimodipine, digital subtraction angiography, case report

## Abstract

Reversible cerebral vasoconstriction syndrome (RCVS) is a cerebrovascular condition marked by the diffuse, reversible narrowing of multiple segments of cerebral arteries. This syndrome predominantly affects women and most commonly presents around the age of 42. It can develop spontaneously or be induced by factors such as the postpartum period or exposure to vasoactive substances. This case report describes an uncommon presentation of RCVS in a 24-year-old woman who developed an intraparenchymal hemorrhage shortly after a spontaneous vaginal delivery, despite the absence of conventional stroke risk factors. Diagnostic imaging with digital subtraction angiography (DSA) revealed multifocal vasoconstriction in the right and left anterior and middle cerebral arteries, along with the vertebrobasilar system, an indication of RCVS. Notably, vasoconstriction improved following intra-arterial nimodipine administration. This case underscores the importance of considering RCVS in postpartum women presenting with headaches and neurological deficits, as timely magnetic resonance imaging, computed tomography (CT) angiography, or brain angiography is critical for accurate diagnosis and optimal management, ultimately reducing the risk of poor prognosis.

## Introduction

Reversible cerebral vasoconstriction syndrome (RCVS) is a cerebrovascular disease defined by variable and reversible narrowing of multiple segments of cerebral arteries, typically linked to endothelial dysfunction and alterations in vascular tone. Over the years, other terms that have been used to describe RCVS have included Call-Fleming syndrome, migraine vasospasm, benign cerebral angiopathy of the central nervous system, pseudovasculitis, and postpartum angiopathy. However, RCVS now serves as the comprehensive term for this condition [1]. The cardinal symptom of this disease is sudden-onset thunderclap headaches, which precede the onset of RCVS with or without other acute neurologic symptoms. The disease is more prevalent in women, with a peak incidence occurring around the age of 42 [2]. The diagnosis of RCVS can be supported by observing segmental narrowing and dilation in at least two different arteries on angiographic examination, provided there is no aneurysmal subarachnoid hemorrhage (SAH). Clinical features such as thunderclap headache (peaking within one minute of onset) or severe recurrent headaches, along with resolution of vasoconstriction within three months, further support the diagnosis [3].

The precise pathophysiology of the syndrome remains unclear; however, these symptoms are believed to be caused by transient disturbances in the regulation of cerebral arterial tone that mainly result from sympathetic overactivation and endothelial dysfunction [4]. The syndrome may develop on its own or in response to certain triggering factors, such as being in the postpartum period or being exposed to vasoactive chemicals [2]. Female reproductive hormones are believed to play a critical role in the development of RCVS. Estrogen has been demonstrated to reduce cerebral tone and inhibit central sympathomimetic activity via prostanoids, endothelial nitric oxide, and other molecular pathways. An imbalance between the pro-angiogenic placental growth factor (PlGF) and the anti-angiogenic soluble PlGF receptor (sFlt-1) is suspected to be a potential trigger for postpartum RCVS [5].

Management of RCVS focuses on symptom control, including pain management and controlling blood pressure. Avoidance of triggers such as certain medications (e.g., vasoactive drugs) and illicit substances (e.g., cocaine) is crucial. In addition to providing management for acute complications such as residual neurological deficits, stopping the triggering factors, and stroke. Nimodipine, a calcium channel blocker, is the first-line drug for the treatment of RCVS. These drugs are administered orally or intravenously and may relieve headaches associated with RCVS. However, there is no evidence that these drugs affect the development of vasoconstriction, stop bleeding, or mitigate ischemic complications linked to RCVS. Balloon angioplasty has also been proposed as a potential endovascular therapy for RCVS [4]. In this case report, we suspected RCVS as the main cause of intracerebral hemorrhage in our patient. Our diagnosis was supported by DSA results showing multiple vasoconstrictions that improved with intra-arterial nimodipine injection. However, the manifestation of RCVS in the form of intracranial hemorrhage is still very rare and here we discussed the best management for our case. A prompt and precise diagnosis can increase the success rate of RCVS therapy.

## Patient and observation

**Patient information:** a 24-year-old woman presented to the hospital with a primary complaint of sudden left-sided weakness a day ago while resting, 2 hours after spontaneous vaginal delivery of her second child. The patient reported a severe headache on the right side of her head with numerical rating scale (NRS) 8 before weakness appeared and vomited about four times without nausea following the headache complaint. Over the following 10 minutes, she had dysarthria and weakness affecting her left arm, left leg, and the left side of her face. There is no seizure, slurred speech, tingling, or decrease of consciousness. Other symptoms are also being denied. She was healthy throughout her pregnancy and in particular was normotensive, without edema, proteinuria, seizures, or headaches. The patient also said that she never had a history of stroke, eclampsia, hypertension, or diabetes mellitus.

**Clinical findings:** the general examination revealed that her overall physical condition and vital signs were normal, with no signs of hypertension. She was fully alert and had no history of decreased consciousness. The neurological examination revealed a central type of left facial palsy and weakness on the left side of her body.

**Figure 1:**
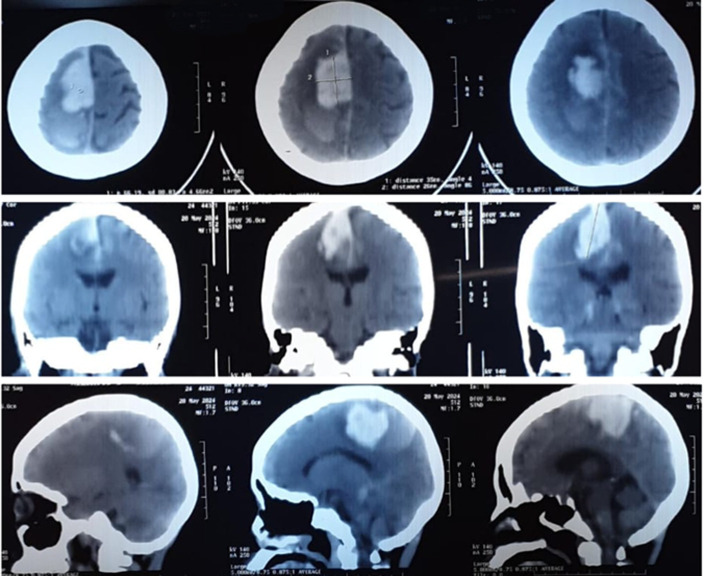
computed tomography of the head without contrast demonstrating a large intraparenchymal hemorrhage centered in the right parietal lobe area

**Figure 2:**
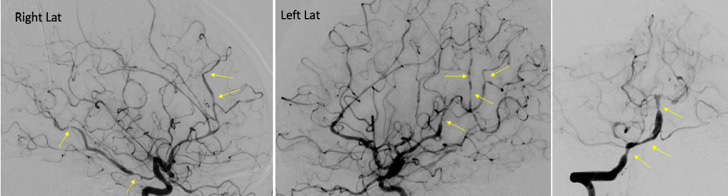
digital subtraction angiography of the right and left anterior and middle cerebral arteries, as well as the vertebrobasilar system, prior to nimodipine injection; multiple vasoconstrictions are observed throughout the visualized arteries (yellow arrows)

**Diagnostic assessment:** the laboratory result revealed high leukocyte and hypoalbuminemia. A significant intraparenchymal hemorrhage in the right parietal lobe area with a volume of approximately 20ml was found from a non-contrast brain CT scan (Figure 1). The patient underwent DSA on May 27, 2024 (Figure 2) and discovered multifocal vasoconstriction in the right anterior and middle cerebral arteries and their distal branches. Multifocal vasoconstrictions were also seen involving the left anterior and middle cerebral arteries and their distal branches, distant from the hemorrhage site. The blood vessels of the vertebrobasilar system also appear vasoconstriction.

**Figure 3:**
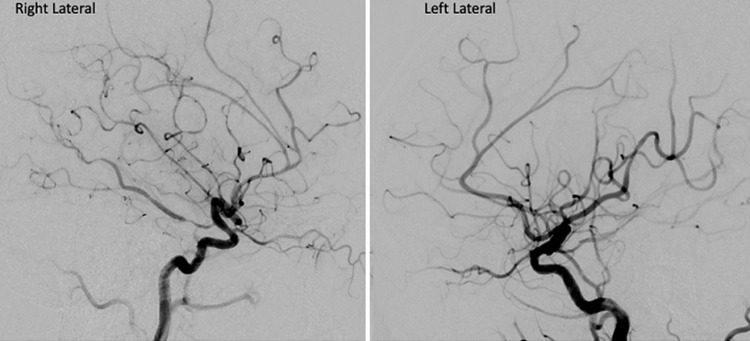
after the injection of intra-arterial nimodipine, digital subtraction angiography revealed a significant improvement in the vasoconstrictions

**Therapeutic intervention:** prompt local improvement in vasoconstriction was noted after selective intra-arterial injection of nimodipine into the right A2 segments of the anterior cerebral artery, posterior communicating artery, and vertebral artery (Figure 3). No signs of aneurysm, branch occlusion, vessel wall irregularity, or arteriovenous shunting were observed. The overall clinical presentation indicated reversible cerebral vasoconstriction syndrome (RCVS). During her hospitalization, she received calcium channel blocker medication and underwent medical rehabilitation exercises.

**Follow-up and outcomes:** the patient was released ten days after the onset because she was clinically stable and did not require any surgical intervention. Approximately one week later, the patient came to the clinic to control her symptoms. She showed improvement in her neurological deficits, including speaking more clearly and being able to lift her left extremity. She could also sit without support.

**Patient perspective:** the patient expressed that she had accepted and understood her illness. She reported significant improvement in her current symptoms and decided to focus on her recovery and caring for her newborn before proceeding with a DSA evaluation.

**Informed consent:** the patient provided written informed consent for the inclusion of their medical details in this case report. The patient has acknowledged that their personal and medical information will be anonymized and used solely for academic and scientific purposes. They have understood the nature of the publication, its significance, and their right to withdraw consent at any time without affecting their medical care. The patient has reviewed the final manuscript and consents to its publication in this medical journal.

## Discussion

This rare and exceptional case emphasizes the peculiar presentation of RCVS, as the patient was initially diagnosed with intraparenchymal hemorrhage that happened soon after giving birth without any associated stroke risk factors. Previous studies have shown that one of the complications of RCVS is intracerebral hemorrhage, with a prevalence rate of up to 44%. However, rather than hemorrhage in the cortex or subcortex, it is noted that convexity subarachnoid hemorrhage is the most prevalent form (20-35%) [4]. The precipitating factor that we found in our patient was the postpartum condition (2 hours after the patient gave birth). Most cases of RCVS can be attributed to identifiable causes, which usually arise during the postpartum period or following exposure to vasoactive substances. Studies have shown that 7-9% of patients develop RCVS during this time. The pathophysiology of RCVS is linked to disruption of the blood-brain barrier, increased vascular tone in the cerebral arteries, and excessive sympathetic activity. The occurrence of transitory central vascular detachment might explain the disease's reversible nature. Multiple vasoconstriction is caused by the innervation of cerebral blood vessels; if this affects small blood vessels, it will lead to reperfusion injury of distal arterioles or rupture of blood vessels, resulting in intraparenchymal hemorrhage [5]. This led to neurological deficit conditions in our patient, such as hemiplegia and severe headaches.

Cerebral catheter DSA is regarded as the gold standard for visualizing vasoconstriction and identifying abnormalities in RCVS cases, particularly in distal vessels, with 2-dimensional DSA offering 100% sensitivity [6]. Additionally, DSA can be used to check for the presence of aneurysms, as aneurysmal SAH must be excluded according to the diagnostic criteria for RCVS [7]. Our patient underwent angiography that showed multifocal vasospasm without any other vascular malformation that improved immediately with [the injection of intra-arterial nimodipine microcatheter in the right A2 segments of the anterior cerebral artery, posterior communicating artery, and vertebral artery. As of right now, there are neither guidelines nor controlled trials pertaining to RCVS; instead, the choice of therapy is based on observational data, expert opinion, and the individual patient's clinical situation [3].

Nimodipine was administered intraarterially to our patients. Although prospective and retrospective studies have demonstrated that nimodipine can alleviate headache symptoms and improve vasoconstriction after intra-arterial vasodilation therapy, recurrence of arterial narrowing has been observed and often necessitates several therapeutic sessions [8]. However, RCVS generally has a favorable prognosis with 90-95% of patients recovering fully within 1-3 months. The vasoconstriction that characterizes the condition typically resolves within this period. Although rare, complications such as stroke, brain hemorrhage, or posterior reversible encephalopathy syndrome (PRES) can occur, potentially affecting the prognosis and leading to long-term neurological deficits. While recurrence of RCVS is uncommon, it is possible, and patients should be monitored for recurrent symptoms [9]. Therefore, further evaluation is necessary to determine whether the nimodipine injection was successful in our case, even though the patient has shown a marked improvement in their clinical condition as a result of treatment. For now, our patient prefers not to undergo an angiography examination because she wants to focus on caring for her baby, and she believes the symptoms, including headaches, have diminished and her limb weakness is starting to improve.

## Conclusion

In order to establish an appropriate diagnosis and treatment, patients with postpartum headache and neurological symptoms, along with evidence of cerebral hemorrhage, should be referred for magnetic resonance imaging, CT angiography, or brain angiography. This is crucial for improving patient outcomes and minimizing the risk of a poor prognosis.
